# Genome divergence and increased virulence of outbreak associated *Salmonella enterica* subspecies *enterica serovar Heidelberg*

**DOI:** 10.1186/s13099-018-0279-0

**Published:** 2018-12-24

**Authors:** Linto Antony, Melissa Behr, Donald Sockett, Dale Miskimins, Nicole Aulik, Jane Christopher-Hennings, Eric Nelson, Marc W. Allard, Joy Scaria

**Affiliations:** 1 Department of Veterinary and Biomedical Sciences, South Dakota State University, Brookings, SD USA; 2 South Dakota Center for Biologics Research and Commercialization, Brookings, SD USA; 3 Wisconsin Veterinary Diagnostic Laboratory, University of Wisconsin, Madison, WI USA; 4 Division of Microbiology, Office of Regulatory Science, Center for Food Safety and Nutrition, U.S. Food and Drug Administration, College Park, MD USA

**Keywords:** *Salmonella Heidelberg*, Outbreak, Genomic epidemiology, SNP, Fimbrial gene, Adhesion, Invasiveness, Multidrug resistance

## Abstract

*Salmonella enterica* serotype *Heidelberg* is primarily a poultry adapted serotype of Salmonella that can also colonize other hosts and cause human disease. In this study, we compared the genomes of outbreak associated non-outbreak causing *Salmonella* ser. *Heidelberg* strains from diverse hosts and geographical regions. Human outbreak associated strains in this study were from a 2015 multistate outbreak of *Salmonella* ser. *Heidelberg* involving 15 states in the United States which originated from bull calves. Our clinicopathologic examination revealed that cases involving *Salmonella* ser. *Heidelberg* strains were predominantly young, less than weeks-old, dairy calves. Pre-existing or concurrent disease was found in the majority of the calves. Detection of *Salmonella* ser. *Heidelberg* correlated with markedly increased death losses clinically comparable to those seen in herds infected with *S.* Dublin, a known serious pathogen of cattle. Whole genome based single nucleotide polymorphism based analysis revealed that these calf isolates formed a distinct cluster along with outbreak associated human isolates. The defining feature of the outbreak associated strains, when compared to older isolates of *S*. *Heidelberg*, is that all isolates in this cluster contained *Saf* fimbrial genes which are generally absent in *S*. *Heidelberg*. The acquisition of several single nucleotide polymorphisms and the gain of *Saf* fimbrial genes may have contributed to the increased disease severity of these *Salmonella* ser. *Heidelberg* strains.

**Electronic supplementary material:**

The online version of this article (10.1186/s13099-018-0279-0) contains supplementary material, which is available to authorized users.

## Introduction

Nontyphoidal *Salmonella* spp. is the leading cause of death and hospitalization due to acquired food borne illnesses in the United States [1]. Episodes of salmonellosis and the resulting annual economic burden is very high in the United States [2]. Livestock and food commodities serve as reservoirs for the disease causing nontyphoidal *Salmonella* spp. Some of these serovars are linked to animal derived food commodities while others are linked to plant derived food commodities [3]. *Salmonella enterica serovar Heidelberg* is one of the common invasive nontyphoidal *Salmonella* associated with greater risk for severe disease [4, 5]. *Salmonella* ser. *Heidelberg* is among the top ten disease causing *Salmonella* serovars in the United States and the third most frequently isolated serovar in Canada [6–9]. Outbreaks of this serovar are attributed mainly to animal derived food commodities with less diversity in isolation sources [3]. This serovar is commonly isolated from eggs and poultry [3, 10–13].

In recent years, the Center for Disease Control and Prevention (CDC) reported six multistate outbreaks caused by *Salmonella* ser. *Heidelberg* in the United States. According to the CDC reports, five of these outbreaks were traced back to either chicken or turkey related products as the source of infection [13–21]. However, a very recent multistate outbreak caused by this serovar has been linked to contact with calves as the source of infection [22]. Epidemiological and laboratory investigation by the CDC identified *Salmonella* ser. *Heidelberg* as the outbreak-causing serovar emerging from dairy calves in Wisconsin which infected people in 15 states between 2015 and 2017. As per the CDC surveillance reports, *Salmonella* ser. *Heidelberg* strains involved in this outbreak are multidrug resistant [22]. During this period, the animal health labs in South Dakota and Wisconsin isolated *Salmonella* ser. *Heidelberg* strains from cases that were causing clinical infections in calves and were possibly related to human outbreaks. This raises the concern of whether *Salmonella* ser. *Heidelberg* has evolved as a bovine adapted lineage with increased colonization and virulence. Previous epidemiological studies on *Salmonella* ser. *Heidelberg* have mostly focused on outbreaks associated with poultry products [23–27]. Most of these analyses were carried out with a limited number of isolates from a small geographical region with a limited number of strains. To overcome the limitations of those studies and to understand the genomic changes that would have contributed to the outbreak-causing bovine origin *S. Heidelberg,* we performed whole genome sequence based comparison of the genomes of the isolates from our labs against more than six hundred *Salmonella* ser. *Heidelberg* strains with diverse host origins and geographical locations. Our analysis includes newly sequenced *Salmonella* ser. *Heidelberg* genomes from outbreak related bovine strains from Wisconsin and South Dakota, and isolates from wild birds. Our whole genome based phylogenomic analysis revealed the presence of a highly divergent multidrug resistant cluster of *Salmonella* ser. *Heidelberg* isolates which have acquired host cell adhesion related virulence functions, subsequently allowing increased bovine colonization.

## Results

### Clinical presentation of calves with *S. Heidelberg*

In the summer of 2016, several Midwestern premises raising bottle-fed calves experienced striking losses in dairy bulls purchased from sale barns. Death rates, usually dictated by neonatal diarrhea which is very common in sale barn calves, increased 5–10-fold. Illness was subsequently found in dairy heifers as well. Disease was apparent even when best practices were used: it affected quality calves given space and comfort, on a good plane of nutrition, with no failure of passive transfer. Sporadic cases were also found dating back to 2014 in our database (see Table 1).

**Table 1:** Features of outbreak associated *Salmonella* ser. *Heidelberg* strains

Assigned name	Biosample ID	Isolation location	Year	Host	Tissue	Lesions	*safABCD*
USA293	SAMN04240660	USA:IA	2015	Bovine	Lung, LN, intestine, kidney	Enteritis; lymphadenitis	+
USA420	SAMN06330648	USA:NE	2016	Bovine	Fecals	No tissues	+
USA520	SAMN03734290	USA:SD	2014	Bovine	Intestine, LN, liver	Ileitis	+
USA521	SAMN03734314	USA:SD	2014	Bovine	Lung, liver, intestine	Enteritis; lymphadenitis	+
USA522	SAMN05781470	USA:SD	2016	Bovine	Intestine, LN, liver, kidney	Villous atrophy	+
USA523	SAMN05781531	USA:SD	2016	Bovine	Intestine, abomasum	Enteritis	+
USA524	SAMN06330649	USA:SD	2016	Bovine	Intestine	No lesions	+
USA526	SAMN06648142	USA:SD	2016	Bovine	Intestine	No lesions, autolysis	+
USA585	SAMN08150382	USA:WI	2016	Bovine	Intestine, spleen, lung	Bronchopneumonia 3/5; lymphoid depletion	+
USA586	SAMN08150383	USA:WI	2016	Bovine	Intestine, LN, lung, bile, liver	Fibrinonecrotic enteritis	+
USA587	SAMN08150384	USA:WI	2016	Bovine	Intestine, Lung, LN	Mild enteritis; lymphoid depletion	+
USA588	SAMN08150385	USA:MN	2017	Bovine	Intestine, LN, lung, abomasum	Enteritis; lymphadenitis	+
USA084	SAMN06231053	USA	2016	bovine	N/A*	N/A*	+
USA089	SAMN05916660	USA	2016	bovine	N/A*	N/A*	+
USA099	SAMN06012648	USA	2016	bovine	N/A*	N/A*	+
USA397	SAMN05752003	USA:MN	2016	bovine	Fecals	N/A*	+
USA460	SAMN05710975	USA:NY	2016	bovine	Intestine	N/A*	+
USA538	SAMN05757192	USA:TX	2011	bovine	Comminuted beef	N/A*	+
USA582	SAMN05852396	USA:WV	2009	bovine	Raw ground beef	N/A*	+
USA114	SAMN06290379	USA	2016	Environmental	Swab	N/A*	+
USA115	SAMN06290384	USA	2016	Environmental	Swab	N/A*	+
USA116	SAMN06290387	USA	2016	Environmental	Swab	N/A*	+
USA117	SAMN06290388	USA	2016	Environmental	Swab	N/A*	+
USA074	SAMN05773458	USA	2015	Human	Stool	N/A*	+
USA080	SAMN05714563	USA	2015	Human	Stool	N/A*	+
USA094	SAMN05773457	USA	2016	Human	Urine	N/A*	+
USA104	SAMN06235037	USA	2016	Human	Blood	N/A*	+
USA107	SAMN07177558	USA	2016	Human	Blood	N/A*	+
USA113	SAMN06290389	USA	2016	Human	Blood	N/A*	+
USA120	SAMN06688282	USA	2017	Human	Stool	N/A*	+
USA088	SAMN05916659	USA	2016	Other	Intestine	N/A*	+
USA303	SAMN07611477	USA:IL	2017	Porcine	Raw ground pork	N/A*	+

A comparison of virulence factors *safA, safB, safC* and *safD* as well as pathologic findings between newly sequenced bovine isolates and other members of cluster bovine

N/A* data not available

### Pathologic findings

In the majority of calves, gross and histologic changes were typical of neonatal calf diarrhea caused by rotavirus, coronavirus or *Cryptosporidium parvum*. Lesions consisted of dehydration with sunken eyes and sticky tissues; mild enteritis due to crypt necrosis or villous atrophy, with the agent of cryptosporidiosis in some cases; mild lymphocytic abomasitis due to irritation of the mucosa secondary to severe metabolic abnormalities (dehydration, metabolic acidosis, electrolyte imbalances); and mild lymphoid depletion in spleen, lymph nodes and Peyer’s patches. In a small number of calves there was severe disease, with typical lesions of enteric salmonellosis: necrosis with fibrin deposition in the superficial small intestinal mucosa, fibrin thrombi in submucosal vessels, and transmural enteritis; also, fibrin, degenerate neutrophils and bacterial emboli in mesenteric lymph nodes; few, randomly scattered foci of necrosis in liver, with bacterial colonies; and severe lymphoid depletion of Peyer’s patches.

### Conclusion of clinicopathologic observations

Predominantly young, < 3-weeks-old dairy calves were the source of *S*. *Heidelberg*. Pre-existing or concurrent disease was found in 9 of 12 younger calves and 3 of 4 older calves, but detection of *Salmonella* ser. *Heidelberg* correlated with markedly increased death losses clinically, comparable to those seen in herds infected with *S.* Dublin, a known serious pathogen of cattle. Salmonellosis was suspected at autopsy or by histopathologic examination in 5 of 12 young calves, and in 0 of 4 older calves, highlighting the importance of bacterial isolation. Since no lesions of salmonellosis were found in older calves, a carrier state cannot be ruled out.

### Single nucleotide polymorphism (SNP) based phylogenetic analysis of *S. Heidelberg*

**Fig. 1:**
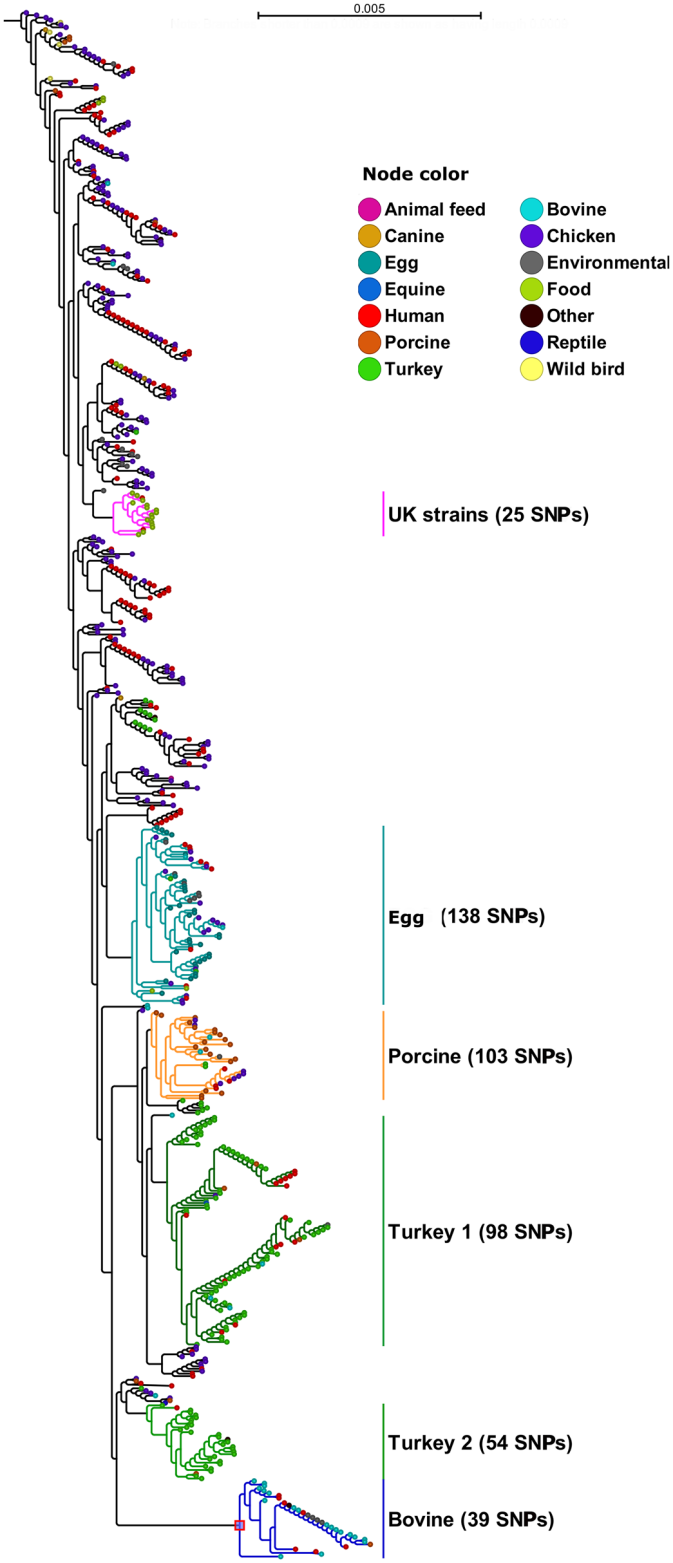
SNP based phylogram of 634 *Salmonella* ser. *Heidelberg* strains from various isolation sources and locations. Phylogenetic tree is arranged in the increasing order of branch length. The entire tree is distributed with a total of 1025 SNP positions. Branches of subtrees in which the major clusters were identified are shown in different colors corresponding to assigned cluster name. Values in bracket indicates the number of SNP positions residing in the corresponding cluster subtree. Cluster names were assigned based on either isolation location category or source country. Node color represents isolation source category of corresponding strain

To determine the possible genomic changes that would have contributed to the increased virulence and its relationship to the strains causing human infections, a reference based SNP analysis of 634 *Salmonella* ser. *Heidelberg* strains was performed with *Salmonella* ser. *Heidelberg* str. SL476 (NCBI accession: NC_011083.1) as a reference genome. This whole genome sequence based SNP comparison revealed 1025 variant positions across the study isolates. Phylogenetic arrangement of genomes in the increasing order of branch length showed clusters of genetically related strains (Fig. 1). Based on our analysis, a chicken isolate from the USA (USA242) with lesser variants and branch length was positioned phylogenetically higher from all other isolates followed by isolate USA488, indicating chicken as the possible evolutionary origin of *Salmonella* ser. *Heidelberg* strains in this outbreak. Recently sequenced wild avian strains by our group (USA 481, USA482, and USA 483), collected in the early 90s, were positioned in the top few clusters closely related to chicken and human isolates.

As the branch length increased, we identified several major clusters in the SNP based phylogenetic analysis of our study isolates (Fig. 1). With increasing branch length, more clusters were differentiated mainly based on isolation sources. An exception was the cluster formed by around 80% of the isolates from the United Kingdom, which contained 25 SNPs that were unique to this cluster. Another interesting observation was that most of the egg isolates remained as a distinct clade having 138 SNPs unique to this cluster. Further down in the SNP tree, more than 50% of isolates from pigs formed a cluster which might be an indication of the expanding host colonization ability of *Salmonella* ser. *Heidelberg* strains. Two of the most divergent clusters, located at the bottom of Fig. 1 in our analysis, primarily originated from cattle, turkey and human cases. The bovine sub-cluster in this analysis was composed of 32 isolates and most were clonal. Defining features of these isolates is given in Table 1. Strain USA460, a bovine isolate, formed an outlier in this cluster indicating bovine origin of all isolates in this cluster. The bovine strains isolated from the animals believed to be originating from Wisconsin closely clustered with human and food isolates. For example, strains USA538 and USA582 originating from ground beef clustered closely with strains USA104 and USA107, which were isolated from human blood. Strains USA114, USA115, USA116 and USA117, environmental isolates from a livestock sale barn in Wisconsin, clustered in the middle of a clonal group of strains that were of human and bovine origin. The outgroup of this cluster was composed of strains that were isolated in the 1980s (USA 141, chicken isolate, 1987; USA128, porcine isolate, 1987) to recent years (Canada008, human isolate, 2012; Canada006, human isolate, 2012) and also from diverse hosts. Therefore, it appears that the strains related to the outbreak associated strains were present in a wider geographical region and a number of hosts other than cattle.

### Virulence mapping of *Salmonella* ser. *Heidelberg* isolates

**Fig. 2:**
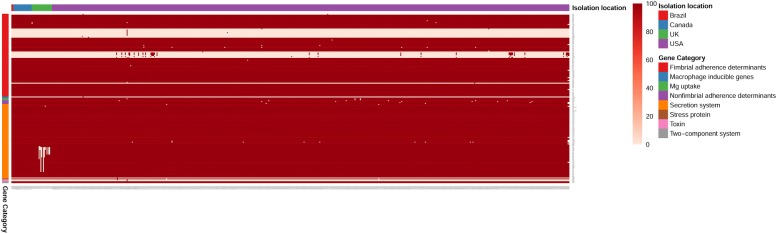
Mapping of virulence determinants in *S*. *Heidelberg*. Heat map showing virulence factors identified in *Salmonella* ser*. Heidelberg* strains under this study. X axis represents sample name based on geographical origin of isolates. Y axis represents virulence gene names. Categories of virulence factors were grouped in to 5 groups. Group legend: 1. Fimbrial adherence determinants, 2. Macrophage inducible genes, Mg Uptake, and Non Fimbrial adherence determinants, 3. Secretion system, 4. Stress protein, Toxin, Two component system. A detailed description of predicted virulence data is available in Additional file 2: Table S2A, B. Color bar represents percentage sequence identity

To understand the virulence repertoire of *Salmonella* ser. *Heidelberg* isolates, we carried out a virulence gene mapping of all 634 genomes. To predict the presence of virulence genes, we performed a local BLAST search of the genomes against virulence gene sequences obtained from the virulence factor database (VFDB) [28, 29]. We grouped 184 identified salmonella virulence genes into four groups (Fig. 2). As expected, the core set of Salmonella virulence genes were conserved among all isolates irrespective of isolation source and their geographical origin. However, isolates in the bovine cluster (Table 1) contained all the 4 fimbrial adherence determinants (*safA, safB*, *safC* and *safD*) of the *Salmonella* atypical fimbriae (*Saf*) gene cluster, while the full cluster was absent in all other isolates compared in this study. A detailed result of the virulence gene mapping across all genomes is given in Additional file 2: Tables 2A, B. Therefore, the virulence mapping of isolates revealed the main hallmark of outbreak associated strains was the presence of *safABCD* operon genes which were absent in all other isolates compared in this study.

### Antimicrobial resistance gene profiling of *Salmonella* ser. *Heidelberg* isolates

**Fig. 3:**
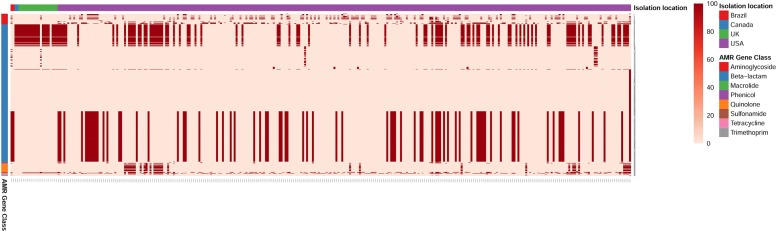
Antimicrobial resistance (AMR) gene profiling of *S*. *Heidelberg*. Heat map showing antimicrobial resistance gene profile of 317 *Salmonella* ser. *Heidelberg* genomes that were predicted for at least one AMR gene. X axis represents sample name based on geographical origin of isolates. Y axis represents class of antimicrobial agents to which the resistance was predicted. A detailed description of predicted AMR is available in Additional file 3: Table S3A, B. Color bar represents percentage sequence identity

To determine the antibiotic resistance genotype among the *Salmonella* ser. *Heidelberg* isolates, we used a comparative genomic analysis approach. We performed a local BLAST search of all 634 of *Salmonella* ser. *Heidelberg* genomes against > 2000 antimicrobial resistance genes in the ResFinder database. A full list of resistance genes identified among these genomes with a minimum 95% sequence identity is given in Additional file 3: Tables 3A, B. Our results show that 50% of the study isolates (317/634) contained least one resistant gene (Fig. 3). Among isolates from chicken, 37% (75/202) were resistant while resistance was 83% (100/120) among turkey isolates. Highest resistance rate (93%) was identified among isolates of bovine origin. Among isolates of human origin, 30% percentage (47/154) contained at least one antibiotic resistance gene. One interesting observation was that, at 95% sequence identity, we could not predict any quinolone resistance genes in poultry isolates except in one turkey isolate (USA525) which was identified with genes *qnrS2* and *qnrS6*. Comparison dataset in this analysis contained isolates dating back to 1980’s. When the resistance profiles of older isolates were compared to recent outbreak causing isolates, we find that the newer isolates contained higher number and type of resistance genes indicating acquisition of more antibiotic resistance among *Salmonella* ser. *Heidelberg* over these years.

## Discussion

Starting from January 2015, outbreaks involving *Salmonella* ser. *Heidelberg* were reported from 15 states in the United States. To date, 56 persons were diagnosed with *Salmonella* ser. *Heidelberg* infections in which 35% [17] were hospitalized and 15% [8] had invasive disease. People who became sick ranged in age from less than 1 year to 72, with a median age of 18, and 33% [18] were less than 5 years of age. Epidemiologic investigations demonstrated that 63% [34] of the infected people had contact with cattle, particularly recently purchased dairy and beef calves. Clinico-pathologic examination revealed pre-existing or concurrent disease in a majority of the calves. Detection of *Salmonella* ser. *Heidelberg* correlated with markedly increased death losses clinically, comparable to those seen in herds infected with *Salmonella* ser. Dublin, a known serious pathogen of cattle. Pathologic findings are intermediate between those caused by the host-adapted *Salmonella* ser. Dublin and disease due to *Salmonella* ser. Typhimurium, which has a broad host range [30]. Laboratory investigations, using polymorphism fragment gel electrophoresis (PFGE) and WGS, linked human isolates as being highly related to the bovine isolates [22]. The goal of this study was to determine the genomic changes that might have contributed to the virulence of *Salmonella* ser. *Heidelberg* strains infecting calves and people.

Whole genome based SNP clustering of human/bovine outbreak strains and other *Salmonella* ser. *Heidelberg* strains from different hosts and geographical regions revealed that human outbreak-associated strains formed a distinct cluster together with calf isolates (Fig. 1). This is not surprising since CDC outbreak investigations already have identified dairy and beef calves from livestock markets in Wisconsin as the likely source of most of the human infections [22]. However, some of the isolates in this cluster predates 2015, which is the beginning of the human outbreak. For example, strain USA520 was isolated from South Dakota in 2014. The WVDL and South Dakota Animal Disease Research and Diagnostic Laboratory have limited isolates prior 2014. The WVDL has an isolate dating back to 2009 that has the same MDR pattern, but the isolate was not available for sequencing. Therefore, it is possible that strains that contributed to this outbreak were present in the Midwest region before 2014.

The outgroup of the outbreak-specific cluster was composed of strains from multiple hosts including chicken, human, porcine, reptile and turkey (Fig. 1). This broad host range of isolations might be an indication that the SNP variations in this cluster may have allowed *Salmonella* ser. *Heidelberg* to better colonize hosts other than poultry. It is possible that these strains may have been the source for the human/bovine isolate causing the current outbreak.

When the virulence gene content was analyzed across all isolates, we find that most of the *Salmonella* virulence genes were present in a majority of the strains. However, the defining feature of the human/bovine outbreak-associated strains was that these isolates contained the presence of *safABCD* operon genes, which were absent in all other isolates (Table 1 and Fig. 2). *Salmonella* contain multiple fimbriae on their surface, many of which play a vital role in attachment with enterocytes to establish and maintain a successful infection. *Salmonella* atypical fimbriae (*Saf*) in *Salmonella enterica* is composed of four contiguous genes encoding the major subunit (*safA*), periplasmic chaperone (*safB*), outer membrane usher (*safC*) and minor subunit (*safD*) [31]. Saf operon is generally absent in *Salmonella* ser. *Heidelberg* [29] while it has been found important for pathogenesis in other serotypes such as *Salmonella* ser. Typhimurium [32]. Transcription of saf fimbrial genes has been found in blood samples from patients infected with *Salmonella* ser. Typhi [33] and *Salmonella* ser. Paratyphi A [34], which is indicative of a role for these genes in the human pathogenesis. Therefore, it is possible that the SNPs associated with the outbreak-causing cluster, together with the acquisition of *Saf* operon genes, could have contributed to the increased virulence of *Salmonella* ser. *Heidelberg* in both calves and people. However, further experimentation in animal models is required to verify this possibility.

Strains in our genomic comparison also included *Salmonella* ser. *Heidelberg* isolates dating back to the 1980s. The antibiotic resistance profile of the old isolates when compared to recent isolates revealed an overall increase in resistance in recent isolates (Fig. 3).

The resistance gene pattern and SNP based clustering did not show any specific correlation. This would indicate that while increased resistance could reduce the options for treatment, acquisition of SNPs in the core genome and virulence genes probably would have been the main driver in expansion of host range and increased infectivity of *Salmonella* ser. *Heidelberg* involved in the outbreak.

## Materials and methods

### Bacterial culture and genomic DNA isolation

Seventeen *Salmonella* ser. *Heidelberg* strains were isolated from cases submitted to the Animal Disease Research and Diagnostic Laboratory, South Dakota State University (ADRDL-SDSU) and Wisconsin Veterinary Diagnostic Laboratory (WVDL) per standard operating procedures. Pathological investigation was carried out following the guidelines set by the American Association of Veterinary Laboratory Diagnosticians. After *Salmonella* isolation and identification, strains were cultured overnight in Luria–Bertani broth. DNA was extracted from 1 mL of the overnight culture using Qiagen’s DNeasy blood and tissue kit (Qiagen, Inc., Valencia, CA) according to the manufacturer’s protocol. Quality of DNA was assessed using Nanodrop One™ (Thermo scientific™, DE). Quantity of DNA was measured using Qubit^®^ 3.0 (Thermo Fisher Scientific Inc., MA) fluorometer and stored at − 20 °C until further use.

### Sequencing library preparation and genome sequencing

Sequencing library was prepared using Nextera XT kit (Illumina Inc. San Diego, CA). Concentration of the DNA samples were adjusted to 0.3 ng/µl prior to processing. After bead normalization, DNA libraries were pooled at equi-molar concentration. Pooled libraries were then sequenced on Illumina Miseq platform (Illumina Inc., CA) using V2 chemistry to generate 2× 250 paired end reads.

### Genome data acquisition and comparative analysis

The raw data files for the seventeen isolates sequenced by our group were de-multiplexed and converted to FASTQ files using Casava v.1.8.2. (Illumina, Inc, San Diego, CA). For comparative analysis, we downloaded publicly available genome sequence data in Fastq format for 617 *Salmonella* ser. *Heidelberg* isolates from the National Center for Biotechnology Information Sequence Read Archive (NCBI-SRA) database using NCBI-SRA tool kit (version 2.8.1-2). To ensure a uniform analytic workflow, we considered only genomes sequenced using the Illumina platform. This constituted 634 genomes in total. Metadata for all genomes are given in Additional file 1: Table S1. Genome data was then imported into CLC Genomics work bench (version 9.5.3, Qiagen bioinformatics) and was further processed. Failed reads as well as reads having length below 200 and above 500 bases were removed. A quality based trimming, with a quality score limit of 0.05 as described by CLC Genomics workbench, was performed to ensure good quality sequence data without any ambiguous nucleotides. Trimmed reads were used for SNP based phylogenetic analysis while assembled genomes were used for virulence gene mapping and antimicrobial resistance gene profiling. For SNP analysis, we used quality trimmed reads mapped to reference genome *Salmonella* ser. *Heidelberg* str. SL476 (NCBI accession: NC_011083.1). This genome was selected as reference as it is a complete genome and has been used as reference for comparative genomics studies of *Salmonella* ser. *Heidelberg* previously [35–38]. For variant calls, fixed ploidy variant detection for bacterial genomes with a minimum 90% variant probability was used. Variant calls and read mappings results were then used to determine the SNP positions and to estimate the consensus sequence. Neighborhood joining method was used to create the phylogenetic tree composed of all 634 genomes. For virulence and antimicrobial gene mapping, we used local databases of *Salmonella enterica* virulence gene sequences available from Virulence Factor Database (VFDB) [28, 29] and ResFinder [39], respectively. Genome assemblies were then used for BLAST searching against these local gene databases with criteria of ≥ 95% minimum sequence identity and ≥ 50% minimum sequence length.

## Additional files

**Additional file 1: Table S1.** Metadata for 634 *Salmonella* ser*. Heidelberg* strains used in this study. Genome sequence data accession and bio sample accession numbers were collected from the NCBI Sequence Read Archive (SRA) for all 634 strains. Assigned name indicates the name given to each strain based on the country from which they were isolated (source country). Isolation location indicates actual location in the source country from which the corresponding strain was isolated.

**Additional file 2: Table S2A.** **List of virulence factors identified in** ***Salmonella*** **ser*. Heidelberg*** **isolates.** Virulence factors identified in 634 *Salmonella* ser*. Heidelberg* strains when all genomes were BLAST searched the against *Salmonella* virulence gene sequences in Virulence Factor Database (VFDB). A) This table represents the tabular view of Fig. 2. Table shows a categorized view of identified virulence factors and the percentage sequence identity corresponding to each isolate. Coloring scheme is based on virulence categories. Values corresponding to each strain represent percentage sequence identity in BLAST search. **Table S2B.** A detailed BLAST search result of virulence genes identified in 634 *Salmonella* ser*. Heidelberg* genomes.

**Additional file 3: Table S3A.** List of antimicrobial resistance genes identified in *Salmonella* ser*. Heidelberg* isolates. Antimicrobial resistance genes identified in 634 Salmonella ser. *Heidelberg* isolates when all genomes were BLAST searched the against resistance gene sequences in ResFinder database. A) Table shows a categorized view of all the AMR genes predicted across 634 *Salmonella* ser*. Heidelberg* genomes. Sample names are given in first row. First and second column represents antimicrobial class and gene name respectively. Coloring is based on Class of antimicrobial agents. Values corresponding to each strain represent BLAST search percentage sequence identity. **Table S3B.** A detailed BLAST search result of AMR genes predicted in 634 *Salmonella* ser*. Heidelberg* genomes.
